# Human Aurora kinase inhibitor Hesperadin reveals epistatic interaction between *Plasmodium falciparum* PfArk1 and PfNek1 kinases

**DOI:** 10.1038/s42003-020-01424-z

**Published:** 2020-11-20

**Authors:** Belinda J. Morahan, Clarissa Abrie, Keith Al-Hasani, Mitchell B. Batty, Victoria Corey, Anne N. Cowell, Jandeli Niemand, Elizabeth A. Winzeler, Lyn-Marie Birkholtz, Christian Doerig, Jose F. Garcia-Bustos

**Affiliations:** 1 Infection and Immunity Program, Monash Biomedicine Discovery Institute and Department of Microbiology, Monash University, Melbourne, VIC 3800 Australia; 2 Faculty of Natural and Agricultural Sciences, Department of Biochemistry, Genetics and Microbiology, Institute for Sustainable Malaria Control, University of Pretoria, Hatfield, 0028 South Africa; 3 Department of Pediatrics, University of California San Diego School of Medicine, 9500 Gilman Drive, MC 0760, La Jolla, CA 92093-0760 USA; 4 Present Address: Department of Diabetes, Monash University Central Clinical School, Alfred Centre, Melbourne, VIC 3004 Australia; 5 Present Address: Illumina, 5200 Illumina Way, San Diego, CA 92122 USA; 6 Present Address: Department of Medicine, University of California San Diego School of Medicine, 9444 Medical Center Drive, MC 0879, La Jolla, CA 92093-0879 USA; 7 Present Address: School of Health and Biomedical Sciences, RMIT University, PO Box 71, Bundoora, VIC 3083 Australia

**Keywords:** Target identification, Chemical genetics

## Abstract

Mitosis has been validated by numerous anti-cancer drugs as being a druggable process, and selective inhibition of parasite proliferation provides an obvious opportunity for therapeutic intervention against malaria. Mitosis is controlled through the interplay between several protein kinases and phosphatases. We show here that inhibitors of human mitotic kinases belonging to the Aurora family inhibit *P. falciparum* proliferation in vitro with various potencies, and that a genetic selection for mutant parasites resistant to one of the drugs, Hesperadin, identifies a resistance mechanism mediated by a member of a different kinase family, PfNek1 (PF3D7_1228300). Intriguingly, loss of PfNek1 catalytic activity provides protection against drug action. This points to an undescribed functional interaction between Ark and Nek kinases and shows that existing inhibitors can be used to validate additional essential and druggable kinase functions in the parasite.

## Abstract (web summary)

Morahan et al. investigate inhibitors of human mitotic kinases in *P. falciparum* and show a resistance mechanism to the drug Hesperadin through an epistatic interaction between the PfArk1 and PfNek1 kinases. This study demonstrates that existing inhibitors can be used to validate additional essential and druggable kinase functions in the parasite.

## Introduction

Apicomplexan parasites such as *Plasmodium* and *Toxoplasma* proliferate using a specialized cell division mechanism termed schizogony, in which the mother cell contents are not fully partitioned between two daughter cells, but rather new cells are assembled around the daughter nuclei after one or several rounds of non-synchronous, closed nuclear divisions, and much of the mother cell cytoplasm is discarded. Unlike in animal cells, there are no conspicuous mitotic spindles with attached condensed chromosomes and the nuclear envelope remains in place (reviewed in ref. ^1^). The spindle pole body (SPB), i.e., the *Plasmodium* organelle homologous to the canonical centrosomal microtubule organizing centre (MTOC), is located within the nuclear envelope, playing a key role in assembling new cells around daughter nuclei: not only does it organize microtubules for chromosome segregation inside the nucleus, it also sends fibres in the opposite direction to mark the pole where the apical invasion complex will be laid down in the emerging daughter cells^2,3^. MTOC duplication signals the onset of mitosis and meiosis in Apicomplexa (the taxon that includes *Plasmodium* spp) and in animal cells as well^4^, with a timing precisely controlled by mitotic kinases. Aurora and ‘Never In Mitosis’ kinase (Nek) families have been described among the regulators of the centrosomal cycle (reviewed in ref. ^5^).

Aurora kinases have long been recognized as potentially useful targets for anti-neoplastic drugs and there are multiple inhibitors at different stages of development (see ref. ^6^ for a review), unlike Nek kinases, which tend to play multiple redundant functions in mitosis and only relatively recently has HsNek2 been implicated in cancer^7,8^. Off-target effects are therefore potentially larger when targeting Neks than other more specialized kinases, because Neks participate in the control of multiple cellular functions involving MTOCs, such as cilliogenesis^9^. In the Aurora kinase class, however, inhibitors are commercially available and represent excellent tools to investigate the function of this kinase family in organisms such as *Plasmodium* *falciparum*, for which genetic tools are scarce, provided evidence can be gathered that the compounds act by inhibiting the predicted orthologous targets in the parasite. Identification of the target(s) in *P. falciparum* of a particularly potent Aurora kinase inhibitor was the object of the present study.

Compounds designed to inhibit human Aurora kinases were identified among the hits of a large phenotypic screen against *P. falciparum*^10^. Given the wide sequence divergence between human and *Plasmodium* enzymes and the promiscuity of many kinase inhibitors, it was conceivable that the lethal target could be an unrelated kinase(s), rather than one of the three Aurora-related kinases (Ark) identified in the *Plasmodium* kinome^11–13^ (reviewed in ref. ^14^). However, if the target(s) were involved in the control of mitosis, the compounds could be useful in elucidating the mechanisms of schizogonic cell division, and at the very least they could be used to identify novel essential pathways in a pathogen in which approximately half the genes have no annotated function. Identification of the molecular targets would also allow development of assays useful in optimizing parasite-specific derivatives, thus enabling novel antimalarial drug discovery based on untapped modes of action. Here we report the results from a forward genetics approach to elucidate the target(s) in *P. falciparum* of several drug-like compounds targeting human Aurora kinases, focussing on the most potent antiparasitic compound, the HsAurB inhibitor Hesperadin. This compound has recently been proposed as an inhibitor of *Leishmania* and *Trypanosoma*^15,16^, although the link between biochemical inhibition and whole-cell activity has not been experimentally established in these systems. We have found that the mode of antiparasitic action of this drug involves a hitherto unknown interaction between PfArk1 and PfNek1, and it may also implicate the 5415-amino acid protein of unknown function PF3D7_1324300.

## Results

### Several human Aurora kinase inhibitors block in vitro growth of *P. falciparum*

Screening of a collection of ~2 million GlaxoSmithKline compounds in a *P. falciparum* growth inhibition assay identified three human Aurora kinase inhibitors among the hits: TCMDC-135395 (Hesperadin), TCMDC-134695 and TCMDC-125873^10^. This number was expanded with four additional commercially available inhibitors with different degrees of specificity towards human Aurora kinases (see references in Supplementary Table 1). Measurement of the concentration that inhibited 50% of growth in vitro (IC_50_) against *P. falciparum* 3D7 and Dd2 showed a range of potencies. Five out of the seven compounds had an IC_50_ in the hundreds of nanomolar, whereas the least potent, Alisertib, was an order of magnitude less potent and Hesperadin was the most potent anti-plasmodial in the set. The latter always displayed an IC_50_ in the tens of nanomolar (Tables 1 and 2, and Supplementary Table 1). All these compounds are described as active against human cells and thus able to permeate biological membranes. Published potencies against human Aurora kinases range over two orders of magnitude (listed in Supplementary Table 1), but they did not correlate with antiparasitic potency in vitro, hinting at differences between the parasite and human targets, although activities against human kinases were not measured in this work in parallel to the anti-plasmodial potency. The compound with the highest antiparasitic potency, Hesperadin, was chosen for further mechanistic studies.

**Table 1:** Mutations found in clonal resistant cell lines and their sensitivity towards Hesperadin.

Genetic background	Clone	PfArk1	PF3D7_1324300	PfNek1	Hesperadin IC_50_ ± SEM (µM)
3D7	Parent	wt	wt	wt	0.07 ± 0.01
	2E1	wt	wt	M66I	2.07 ± 0.12
	3E6	wt	wt	M66I	2.32 + 0.24
	6G7	V59L	S2393T	wt	4.20 ± 0.01
Dd2	Parent	wt	wt	wt	0.03 ± 0.01
	8E6	wt	wt	S854Stop	2.03 ± 0.06
	1F9	wt	wt	G143D	3.55 ± 0.17
	5G8	wt	wt	A246G	4.53 ± 0.21

Data are averages of three biological experiments, each with three technical replicates, plus or minus the SEM.

**Table 2:** Hesperadin IC_50_ of PfNek1 truncations.

Genetic background	Strain or clone	Hesperadin IC_50_ (µM)
3D7 Nek1:HA	Drug-sensitive control	0.005 ± 0.002
3D7 Nek1D(854-1057):HA	1G2	1.9 ± 0.4
3D7 Nek1D(854-1057):HA	2F11	1.8 ± 0.5
Dd2 Nek1 (854 stop)	Original resistant mutant	2.03 ± 0.06

Data are averages of three biological experiments, each with three technical replicates, plus or minus the SEM.

### Hesperadin blocks nuclear division in *P. falciparum*

The effect of Hesperadin on *P. falciparum* mitosis was tested by synchronizing parasites at the G1/S transition point using the polyamine depletion method (>95% synchronicity within 3–4 h age window)^17^. Parasite cultures released from the cell cycle block in the presence (0.5 µM, 10× IC_50_ for the 3D7 clone used in the experiment) or absence of Hesperadin were followed for 48 h and analysed at 6 h intervals for parasitemia, nuclear morphology and cell cycle stage, using confocal microscopy and flow cytometry.

**Fig. 1:**
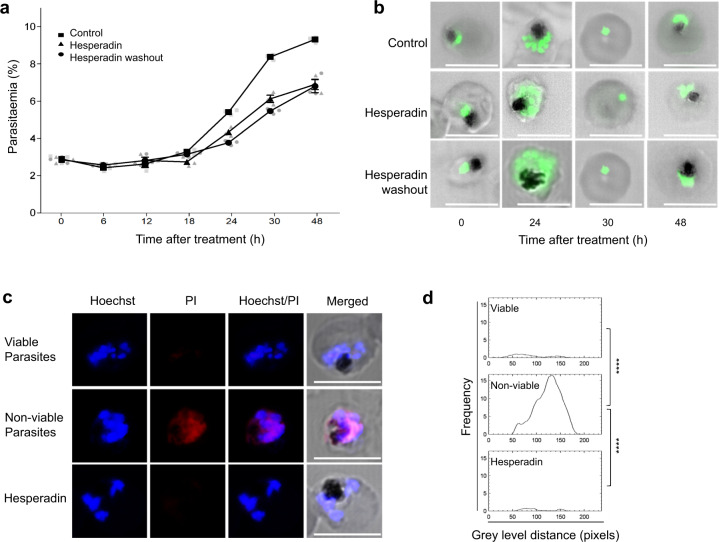
Effect of Hesperadin on proliferation and viability of synchronized parasites. Intraerythrocytic *P. falciparum* 3D7 control parasites (3% parasitaemia, 5% haematocrit) were treated with 500 nM hesperadin and sampled every 6 h after hesperadin treatment. **a** Growth curves of parasite cultures untreated (control, squares), treated with 500 nM Hesperadin (Hesperadin, triangles), and similarly treated but washed free of drug after 18 h (Hesperadin washout, circles). Grey symbols represent averaged counts from three independent biological experiments, each scored as three technical replicates. The average of three experiments is shown as black symbols, with a bar indicating the SE, except when the symbol is larger than the error bar. Data are available in Supplementary Data 1. **b** SYBR green staining of parasites from **a**. Note the stained material between nuclei in drug-treated parasites. **c** Fluorescence micrographs of viable (top row), methanol-treated (middle row), and Hesperadin-treated parasites (bottom row), stained with the intercalating dyes Hoechst 33258 and propidium iodide. **d** Quantification of propidium iodide fluorescence in the images shown in **d**. Significant differences were calculated using two-tailed equal variance Students *t*-test, ****P < 0.0001. Images were captured with a Zeiss LSM 880 Confocal Laser Scanning Microscope (LSM). Scale bars apply to all micrographs and correspond to 5 µm.

**Fig. 2:**
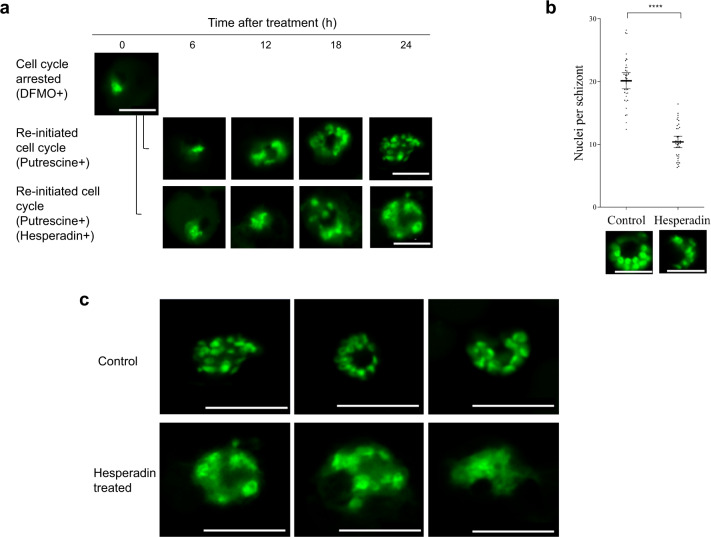
Nuclei number and aberrant nuclear morphology of parasites after Hesperadin treatment. Intraerythrocytic *P. falciparum* 3D7 parasites were synchronized with DFMO and then allowed to re-initiate their cell cycle by addition of putrescine, in the presence or absence of Hesperadin (24 h treatment). **a** Parasites were sampled every 6 h after hesperadin treatment and life cycle progression monitored through SYBR Green I fluorescence microscopy using a Zeiss LSM 880 Confocal Laser Scanning Microscope (LSM). **b** Control and hesperadin-treated parasite nuclei development was quantitatively assessed through SYBR Green I fluorescence microscopy. Inter-nuclei distances were determined for a minimum of ten nuclei. Significant difference was calculated using two-tailed equal variance Students *t*-test, ****P < 0.0001, *n* = 36. Error bars represent 95% CI of the mean. Scatter plots were generated using GraphPad Prism version 6.01 software. Data are available in Supplementary Data 1. **c** Representative pictures of morphological abnormalities observed in Hesperadin-treated nuclei for three individual schizonts each. Scale bars apply to all micrographs and correspond to 5 µm.

**Fig. 3:**
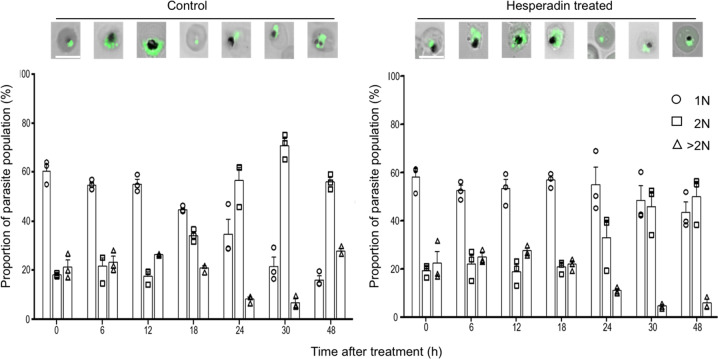
Flow cytometry analysis of SYBR green staining material in asynchronous parasites untreated (control) or treated with Hesperadin. Fluorescence microscopy of representative parasites from each time point is shown on top of the groups of bars. The relative ploidy of individual parasite counts included in each bar is shown on the legend to the right, assuming early rings are 1N (circles). Parasites with twice the DNA content (2N) are indicated by square symbols and counts of parasites with larger ploidy are indicated by triangles. Each symbol corresponds to an independent biological experiment and it is the average of three technical replicates. Bars are the average of the three experiments with the SEM.

**Fig. 4:**
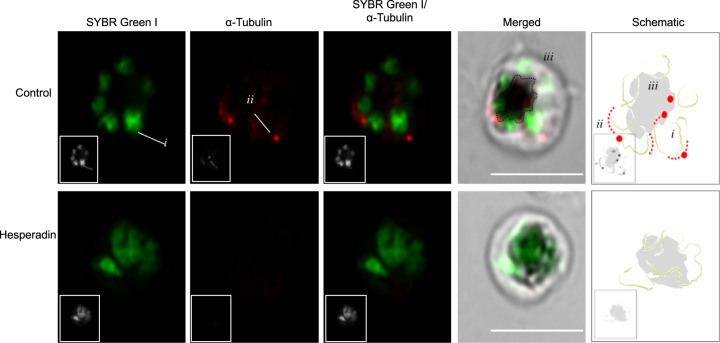
Relative positions of SYBR green and AlexaFluor 647-conjugated anti-α-tubulin-stained material in untreated (control) and Hesperadin-treated parasites. The column labelled ‘Merged’ is a superposition of the fluorescence images in the red and green channels with a differential interference contrast image of the same parasite. The column labelled ‘Schematic’ is an interpretation of the relative positions of the nuclear material (i), the mitotic spindle (ii) and haemozoin crystals in the food vacuole (iii); actual images of nuclear and spindle material are also indicated for a single daughter cell in the control row. Images are representative of at least seven parasites evaluated per sample. Black and white versions of the colour pictures have been added as inserts. Scale bars apply to all colour micrographs and correspond to 5 µm.

Parasitemia measured by flow cytometry of SYBR Green-stained cells showed that drug treatment irreversibly reduced the number of parasites able to re-invade (Fig. 1a), with treated schizonts displaying an abnormal morphology (Fig. 1b). This effect was not due to overt cellular damage, as parasites remained able to exclude propidium iodide, unlike parasites rendered non-viable with a methanol wash (Fig. 1c, d). The reduction in parasitemia followed a ~50% reduction in the total number of nuclei per schizont after Hesperadin treatment (Fig. 2a), which could be quantified with statistical significance after 24 h (Fig. 2b). In addition to this reduction in number, the nuclei of drug-treated parasites also displayed morphological abnormalities, appearing bloated, sometimes multi-lobbed, with SYBR Green-stained material extending between them (Fig. 2c), probably representing DNA that failed to get packaged inside daughter nuclei. These results are suggestive of a dysregulation of the nuclear division process, whereby normal DNA replication and assembly of daughter nuclei is compromised. This is consistent with results from the analysis of population ploidy by flow cytometry. The fraction of individual cells with 2N and higher DNA content was lower in drug-treated parasites, and diverged from that of the untreated culture starting at 18 h after cell cycle re-initiation (Fig. 3). This morphologically aberrant and lower yield nuclear division correlates with a striking absence of nuclear α-tubulin staining in Hesperadin-treated schizonts (Fig. 4), indicating that the drug affects the ability of the SPB to organize the mitotic spindle and hence proper chromosome assembly and replication.

### PfArk1 and PfNek1 mutations found in *P. falciparum* cell lines resistant to Hesperadin

To investigate the lethal target(s) of Hesperadin in *Plasmodium*, a resistant mutant selection experiment was carried out using two different genetic backgrounds, the reference 3D strain and the multi-drug-resistant Dd2.

Prior to the experiment, the genetic heterogeneity of the two parental lines was reduced by isolating single clones through limiting dilution. Selection was applied on four 10-ml cultures per strain, containing ~2 × 10^8^ parasites each, passaged for up to four months in the presence of 0.4 μM Hesperadin (10× IC_50_). Dd2 cultures were the first to be exposed to the drug and three of the four flasks produced stably resistant parasite populations, from which drug-resistant clonal cell lines were obtained by limiting dilution. Two clones from each culture were retained but only parasites from different flasks were considered independent mutants. IC_50_ values were determined and whole-genome sequencing (WGS) performed on the independent isolates plus their parental clone. On the 3D7 arm of the experiment, four similar cultures were subjected to the same drug pressure. Resistant parasites emerged from one culture in time for a single clone to be sequenced together with the three resistant Dd2 clones obtained earlier. Half a month later, two additional 3D7 cultures produced drug-resistant growth. Only targeted sequencing of the candidate genes identified through WGS in the earlier resistant clones was carried out in these late-arising 3D7 clones. In total, three cultures from each genetic background produced drug-resistant parasites, giving an aggregated apparent frequency of one pre-existing resistant mutant cell per 4 × 10^9^ starting parasites. The assumption that all Hesperadin-resistant growth in each flask stemmed from a single mutant parasite is supported by the finding that the two clones isolated from each flask by limiting dilution had the same base change. Table 1 illustrates the IC_50_ of Hesperadin against the resistant clones. An ~100-fold increase had occurred in a single selection step. Interestingly, preliminary evidence indicates that Hesperadin-resistant lines do not show significant cross-resistance to other Aurora kinase inhibitors in Supplementary Table 1, suggesting that the resistance mechanism and possibly the mode of anti-plasmodial action are unique to Hesperadin and not generalizable to the other human Aurora kinase inhibitors tested.

The full genomes of the first four resistant clones and of their respective parental strains were sequenced using Illumina chemistry to a mean coverage of 68–92% with >97% the genome covered by five or more reads (Supplementary Table 2). Data were analysed using a custom-made automated bioinformatics pipeline^18^ as described in detail in the ‘Methods’ section. Results included quality-weighted single-nucleotide polymorphism (SNP) calls and copy number variations. No significant copy number changes were detected. Excluding polymorphic gene families, the highest quality non-synonymous SNPs absent from the parental strains were found in two proteins (PfArk1/ PF3D7_0605300 and PF3D7_1324300) in the 3D7-derived isolate 6G5, and in a single protein (PfNek1/ PF3D7_1228300) in all three Dd2-derived isolates (Table 1 and Supplementary Table 2).

All genetic changes displayed in Table 1 were confirmed by directed sequencing. Targeted sequencing of the identified loci in the two late-arising 3D7 clones, 2E1 and 3E6, revealed a mutation in PfNek1 only and parental sequences at both PfArk1 and PF3D7_1324300. Therefore, only one of the six Hesperadin-resistant clones carried a mutation in PfArk1, while five carried a mutation in PfNek1, a “Never in Mitosis A” (NIMA)-related kinase that is phylogenetically unrelated to the Aurora family^14^. Four different mutations were detected in *Pf*Nek1: M66I in two 3D7-derived clones (from different nucleotide changes) and G143D, A246G and S854STOP in the Dd2-derived clones.

PfArk1 is a 353-residue kinase with 28% sequence identity to HsAurB, the main target of Hesperadin in humans^19^. Protein sequence alignment with vertebrate AurB using Clustal Omega^20^ at the EBI server^21^ and inspection of the published X-ray structure of the *Xenopus* ortholog bound to Hesperadin (PDB ID: 2BFY^22^) indicated that the alanine residue in all vertebrate AurB that aligns with the V59L mutation in *Plasmodium* is located in close proximity to bound Hesperadin. The one-carbon length extension introduced by the mutation could exert a steric hindrance on Hesperadin binding, suggesting a mechanism for resisting the drug. Unlike PfNek1, it has not been possible to express PfArk1 as an active recombinant protein, which has hampered biochemical characterization of its role in Hesperadin’s mode of action.

### PfNek1 role in Hesperadin’s mode of action

**Fig. 5:**
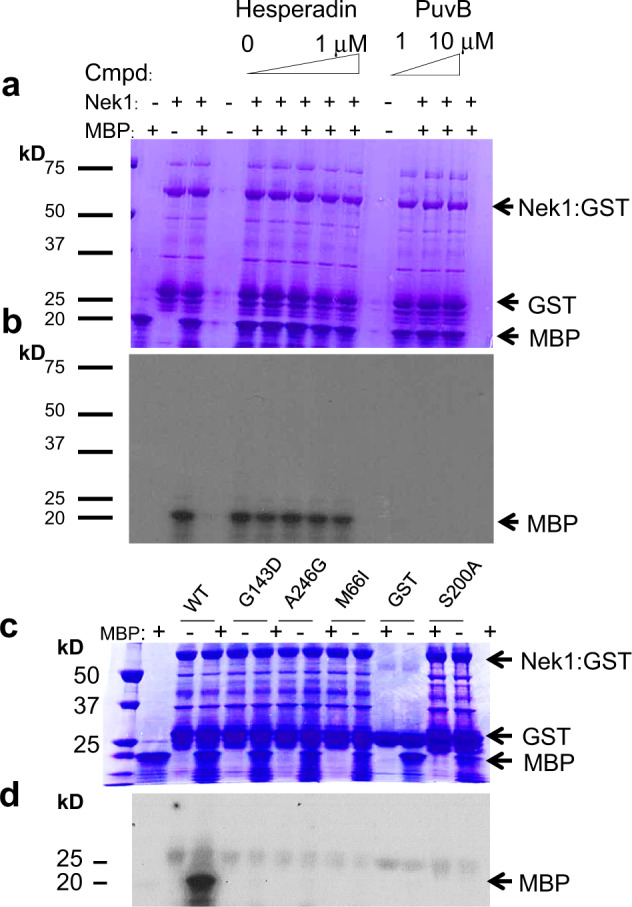
Protein kinase activity of GST:PfNek1 fusion proteins. **a** Coomassie gel of wild-type Nek1 incubated with MBP and the indicated inhibitors (PuvB, purvalanol B). **b** Autoradiogram of the gel in **a**, showing MBP phosphorylation. **c** Coomassie gel of wild-type Nek1 (wt), mutant Nek1 (mutations G143D, A246G, M66I and inactive control S200A indicated) and GST-only negative control. **d** Autoradiogram of the gel in **c**, showing MBP phosphorylation. Full-length gel and autoradiography for panels **c** and **d** are shown in Supplementary Fig. 3.

Despite the low sequence conservation between PfArk1 and PfNek1, it is conceivable that Hesperadin is able to inhibit both essential kinases. PfNek1 is a larger, 1057-residue protein with 27% sequence identity to PfArk1 in the catalytic domain that has been detected in the nucleus and cytoplasm of the parasite^23^. Three of the four mutations detected in resistant cell lines fell in the N-terminal catalytic domain, suggesting they might affect inhibitor binding. The fourth one is a very interesting C-terminal stop codon that would eliminate the second predicted coiled-coil domain^14^. These coiled domains are a common feature of NIMA-related kinases and in the closest human ortholog, HsNek2 (39% sequence identity in catalytic domain), they play an essential role in protein dimerization and activation^24^. This may explain the mechanism of resistance afforded by the nonsense mutant in light of the results obtained with the catalytic domain mutations (see below). The PfNek1 kinase domain, amino acid residues 1–432, has been expressed as a recombinant GST fusion and shown to phosphorylate MBP in vitro^25^. The three point mutations found in this domain in Hesperadin-resistant cell lines (Table 1) were introduced into a PfNek1 expression construct. Recombinant mutant and wild-type proteins were expressed in *Escherichia coli*, semi-purified, concentrated on glutathione beads and used in kinase assays to assess their protein kinase activity and sensitivity to Hesperadin inhibition. Unexpectedly, the catalytic activity of wild-type PfNek1 was not inhibited by Hesperadin, although it remained sensitive to a different inhibitor, Purvalanol B^25^ (Fig. 5a, b). Even more unexpectedly (given PfNek1genetic essentiality^23^), all three mutants were catalytically inactive, just like the K44M and S200A mutants described  in ref. ^25^ (Fig. 5c, d and Supplementary Fig. 3), implying that at least in blood stages the essential function of PfNek1 is not dependent on kinase catalytic activity, but perhaps on its ability to serve as a bridge or scaffold for other signalling components. Whatever its precise function, the results strongly indicated that loss of PfNek1 catalytic activity allows cell division in the presence of 68-times the wild-type IC_50_ of the inhibitor, a concentration at which its primary target would be 99% inhibited.

### Deletion of the carboxyl terminus of PfNek1 is sufficient to provide resistance to Hesperadin in *P. falciparum* 3D7

**Fig. 6:**
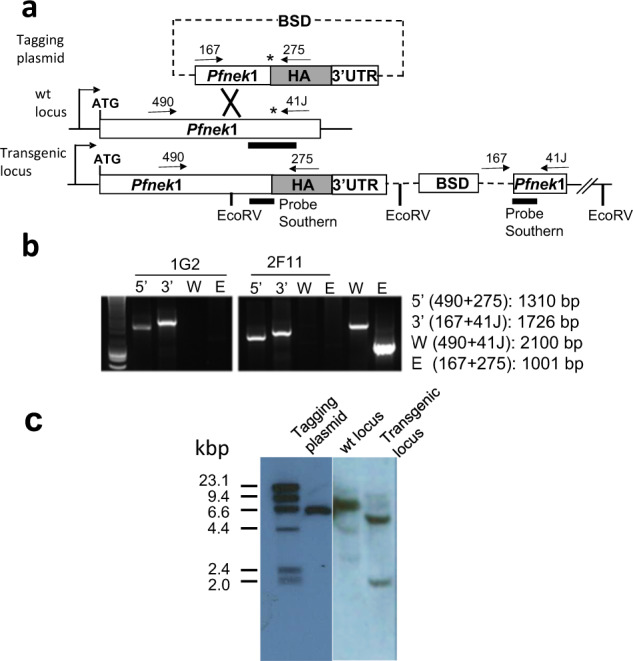
Insertional mutagenesis of the chromosomal *Pfnek1* locus to truncate the kinase at amino acid position 854. **a** Scheme showing the integrative plasmid, the wild-type locus and the expected integration event, PCR primers used to test transductants and the DNA probe for the Southern blot in **c**. **b** PCR results for transductant clones 1G2 and 2F11, showing the expected size bands for the 5′- and 3′-junctions. Bands from the wild-type locus (W) and the free plasmid (E) are shown for reference. **c** Southern blot of EcoRV digested genomic DNA from clone 1G2, hybridized to the probe illustrated in **a** and showing the desired disruption of the wild-type locus.

The most direct way to link the individual mutations to Hesperadin resistance would be to introduce them into a wild-type background and measure drug sensitivity of the transfectants. This is difficult to achieve in the 5′-regions of genes and attempts to perform the experiment using a CRISPR-Cas9 approach failed. In contrast, the C-terminal S854STOP nonsense mutation found in clone 8E6 is easier to introduce by a single cross-over event, using approaches developed to produce tagged proteins through C-terminal fusions. A tagging vector was constructed using the pCAM-BSD-HA backbone^26,27^, into which 1027 bp of *Pfnek1* sequence immediately 5′ to the S854STOP mutation were inserted. The resulting plasmid contained the coding sequence for 342 internal PfNek1 amino acids ending at residue number 853, fused in-frame to an HA epitope (Fig. 6a). This construct was transformed into a wild-type 3D7 cell line in duplicated experiments and integrants were selected by two 1-week cycles on and off Blasticidin. Two individual clonal cell lines were isolated from each transformation by limiting dilution in the presence of 10x IC_50_ Hesperadin, to select for integrations able to provide drug resistance. PCR amplification of the insertion junctions in one clone from each transformation produced the expected genomic bands, without visible contributions from plasmid or wild-type genomic sequences (Fig. 6b). Clone 1G2 was chosen for further analysis and the DNA sequence at both ends of the new PfNek1:HA open reading frame (ORF) was determined, including the PfNek1-HA junction. It showed the expected N- and C-terminal ends for the desired truncated PfNek1 fused to the HA tag (see scheme in Fig. 6a). We attempted to test expression of the truncated protein by Western blot. Unfortunately, three different anti-HA antibodies were not able to detect the shortened protein or a control full-length HA-tagged version in total parasite lysates, probably because of low PfNek1 abundance. We did however obtain Southern blot evidence that the wild-type *nek1* locus had been disrupted, without duplication of the wt gene. Total genomic DNA was digested with EcoRV and a blot of the digest was probed with a 500 bp fragment spanning the site of the nonsense mutation, which should have been split by the HA tag in the deletion construct. The probe detected a single restriction fragment in the wild-type genomic DNA as expected. This EcoRV band migrated at approximately 7 kbp and appeared split into 5 and 2 kbp fragments in the transfected cell line (Fig. 6c), showing that there was only one *nek1* locus in the transfectant line and that it had been changed at the correct position.

One parasite clone from each of line carrying a truncated PfNek1 C-terminus was tested for sensitivity to Hesperadin. IC_50_ was measured in comparison to the original Dd2 resistant cell line and a 3D7 strain expressing an HA-tagged full-length PfNek1, as a fully sensitive control. Data (Table 2) show that truncation of PfNek1 at amino acid 853 in a 3D7 background is sufficient to confer Hesperadin resistance at the same level as in the original Dd2 mutant. This result, together with the lack of phosphorylating activity in the mutant PfNek1 catalytic domains (Fig. 5), can be interpreted to mean that a kinase lacking the predicted distal C-terminal coil may be unable to dimerize, or participate in some key protein complex, making the C-terminal deletion functionally equivalent to a loss of catalytic activity. Dimerization itself does not seem required for kinase activity, as the isolated catalytic domain is active when expressed as a recombinant protein^25^ (Fig. 5 and Supplementary Fig. 3).

**Fig. 7:**
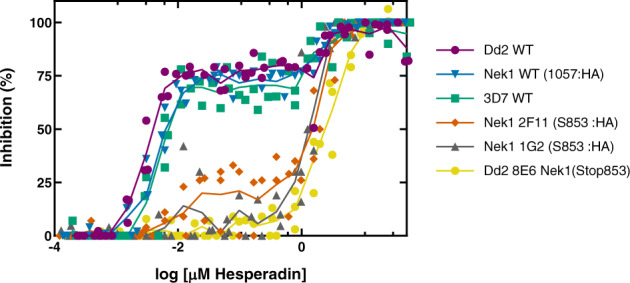
Dose–response curves of wild-type and PfNek1 mutant *Plasmodium* strains treated with Hesperadin. Strains analysed are indicated in the legend. They were wild-type laboratory strains (Dd2, 3D7), a strain bearing a complete PfNek1-HA fusion (Nek1 1057:HA), a Hesperadin-resistant spontaneous mutant (Dd2 8E6) and two transfectant clones with a Nek1 truncation at amino acid 853 (2F11 and 1G2). All data points are shown, slightly offset along the *x*-axis to avoid overlap. Lines connect the average inhibition values at each Hesperadin concentration. Data are available in Supplementary Data 1.

Figure 7 shows the data points generated when measuring the IC_50_ values listed in Table 2, with the addition of a wild-type 3D7 reference strain. Although Table 2 values were generated from an interpolated monophasic sigmoidal curve in Graphpad Prism, visual inspection of the data strongly suggests that the dose/response curves are biphasic for all sensitive strains and the two clones with truncated PfNek1. IC_50_ values on the table were generated by the software from the first transition in the dose–response curve in the sensitive strains and from the second transition in the resistant clones. This type of dose–response suggests the existence of different targets with varying sensitivities to the drug, and PfNek1 inactivation appears to enable bypassing the inhibition of the most sensitive process.

## Discussion

Aurora kinases are typically involved in controlling division of centrosomes and kinetochores during mitosis and meiosis in higher Eukaryotes (reviewed in^28^) and there are three essential *Plasmodium* orthologues, PfArk1, PfArk2 and PfArk3^12,23,29^. Here we show that a number of ATP-competitive inhibitors targeting human Aurora kinases with varying selectivity are also inhibitors of *P. falciparum* growth in vitro.

We used a forward genetic approach to target identification, a method that has been well-tested in the context of antimicrobials, and that has also delivered results in *P. falciparum* (see ref. ^30^ for a discussion). Single-step selection of spontaneous mutants resistant to 10× IC_50_ of the most potent anti-plasmodial compound in the set, Hesperadin, allowed isolation of six independent resistant cell lines; three in the 3D7 background and another three in the multi-drug-resistant Dd2 strain. WGS identified single high scoring mutations in *Pfark*1 (one mutation, together with a mutation in PF3D7_1324300) and in *Pfnek*1 (four independent mutations). Both PfArk1 and PfNek1 are essential for *P. falciparum* asexual proliferation in red blood cells (RBCs)^23,29,31^. They display little homology outside the kinase-defining motifs and thus are not expected a priori to be inhibited by the same highly selective drug, even if it is an ATP-competitive inhibitor. In addition to the two kinases, the other protein found mutated, PF3D7_1324300 is a large protein (659 kDa) with an abundance of positively charged residues (pI = 9.37). Annotations in PlasmoDB (release 45) do not mention functional motifs other than five predicted transmembrane domains, plus two phosphorylation sites and four acetylated lysine residues^32^. The COILS software^33^ predicts a high probability of multiple coiled-coil domains in the N-terminal half of the protein, which would be compatible with a scaffolding function within a multi-protein complex. A fragment of this protein was found to interacted with PF3D7_0309300 (PFC0390w), a protein of unknown function, in a large yeast two-hybrid protein interaction screen^34^. Whether the PfArk1 mutation needs to be genetically linked to the PF3D7_1324300 mutation for the resistance phenotype to emerge is unknown at present, although the change in the latter protein (S2393T) is suggestive of a kinase substrate site. This position is however not annotated as a phosphopeptide in PlasmoDB (release 45)^32^ and the contribution of this mutation to Hesperadin resistance, as well as the function of PF3D7_1324300 require additional investigation. It is nevertheless noteworthy that large coiled-coil proteins, such as PF3D7_1324300, are known substrates or binding partners/scaffolds for Aurora and Nek kinases at centrosomes and kinetochores^35–37^.

PfArk1 associates transiently with the periphery of dividing nuclei in parasites^12^ and it is therefore assumed to regulate SPB division or kinetochore separation, similar to the role of mammalian Aurora kinases. Despite trying several different approaches, we have been unsuccessful so far in generating recombinant active PfArk1 to directly test its sensitivity to Hesperadin. Pulldowns from a *P. falciparum* strain expressing green fluorescent protein-tagged protein^12^ were also inactive, hindering biochemical testing. Interestingly, however, the Hesperadin-resistant mutants did not appear cross-resistant to the other human Aurora inhibitors tested, suggesting that Hesperadin has a unique mode of action in *P. falciparum*. It has been previously shown that the three *Pfark* loci can be genetically tagged but not disrupted^29^. This is evidence for an essential role in the blood stages of the parasite and it is possible that other inhibitors of human Aurora kinases may be targeting PfArk2 or PfArk3, without excluding non-homologous kinases.

The surprising observation that mutations in PfNek1 lead to Hesperadin resistance raises several important questions. The first one is how a genetically essential kinase can lose catalytic activity without affecting parasite viability. The simplest answer may be that its essential function does not require phopshotransfer catalysis, but perhaps just protein-protein interactions, at least in blood stages. After all, apicomplexan genomes are known to encode essential pseudokinases (cf. refs. ^38,39^). Nek kinases are described to have redundant functions as components of multi-protein complexes involved in centrosome maturation and division (see refs. ^5,40^ for reviews). A second important question is how loss of PfNek1 activity could overcome the consequences of PfArk1 inhibition, (assuming PfArk1 is indeed the lethal target of the inhibitor). This would point to a hitherto unknown epistatic interaction between the two kinases in *P. falciparum* that is currently being explored. One hypothesis is that PfNek1 activity mediates one of several mechanisms to prevent untimely division of centrosomes. If PfArk1 activity were necessary to relieve repression by PfNek1, it would explain the results presented above. In a wild-type cell, compounds inhibiting PfArk1 would repress nuclear division by allowing the unrestrained action of PfNek1, whereas inactivation of PfNek1 would make nuclear division independent of PfArk1 activity and, consequently, resistant to inhibitors of this latter kinase. This model predicts that blocking PfNek1 activity would actually antagonize the effect of a selective PfArk1 inhibitor on parasite proliferation, at least in the blood stages. Purvalanol B cannot be used to test the model due to its low specificity^41^. In apparent agreement, of the non-synonymous SNPs in the genetic loci encoding PfArk1 and PfNek1, listed in MalariaGen^42^, there is only a single and very conservative E to D change in PfArk1, whereas multiple PfNek1 variants have been detected, several of which localize to the catalytic domain, although there is no experimental evidence that they affect phosphor transfer activity. None of the published SNPs coincide with the mutated nucleotide positions described here.

Hesperadin has recently been reported to inhibit Aurora kinases and cell division in other parasitic protists, whereas Aurora inhibitors with greater biochemical potency against the parasite enzyme in in vitro kinase assays had no (or much lower than expected) whole-cell activity^15,16^. This could be interpreted as a lack of cell penetration or loss of drug through efflux; however, our findings of (i) the apparent lack of cross-resistance between various Aurora kinase inhibitors and (ii) epistasis between mitotic kinases, allow for a variety of possible modes of action. Identification of the targets involved and detailed elucidation of their interactions would open the way to the development of selective antiparasitic drugs devoid of significant activity against human targets, taking advantage of the large body of knowledge being accumulated on kinase inhibition for other indications. However, as shown here, inhibition of the Ark1/Nek1 pathway can select for spontaneous resistant mutants at a readily measurable frequency in vitro, and compounds will have to be used in combination to prolong their usefulness, although this is usually the case for most antimicrobials.

## Methods

### Parasite cultures

Parasites were cultured in vitro in complete RPMI medium containing Albumax II (lipid-rich bovine serum albumin) (Gibco) (0.5% w/v), gentamycin (Pfizer) (10 µg/ml), O + RBCs to 4% haematocrit (Australian Red Cross). Cultures were gassed with 1% O_2_, 5% CO_2_ and 94% N_2_ (1 s/ml of culture) and kept closed airtight at 37 °C. Where required, cells were cultured under drug selection with either 5 nM WR99210 (Jacobus Pharmaceuticals) or 5.4 µM Blasticidin S-HCl InvivoGen).

### Growth inhibition assays

The concentration of compound able to reduce parasite growth by 50% (IC_50_) relative to an untreated control was measured by making 2-fold serial dilutions of compounds in the complete RPMI medium described above. Compounds at 10 mM in dimethyl sulfoxide were added (1.5 µl) to the first well in the dilution series of a 96-well plate, containing 300 µl of parasite culture at 0.25% parasitemia in 2% haematocrit. This gave a 50 µM starting concentration that was diluted 2-fold into successive wells containing 150 µl of the same parasite culture, until a concentration of 0.76 nM compound was reached. Triplicate no-treatment control wells contained 0.5% dimethyl sulfoxide (DMSO), while no-grow control wells received a mixture of 50 µM chloroquine and 50 µM artemisinin (Sigma). Plates were incubated as above for 72 h and the activity of *P. falciparum* lactate dehydrogenase in each well after the incubation period was quantified as a correlate of parasite numbers as previously described^10^.

### In vitro parasite synchronization, flow cytometry and fluorescence microscopy

In vitro-cultivated early ring-stage (within 12 h post-invasion) *P. falciparum* parasites (3D7 or NF54 strains, 1% haematocrit, 1% parasitaemia) were tightly synchronized in early trophozoite stages (18–22 h post-invasion, >95% synchronicity, max 4 h window in age range) by treatment with DL-α-difluoromethylornithine (DFMO, 2 mM) for 24 h. DFMO-induced arrest was reversed with the addition of 2 mM putrescine either in the absence or presence of Hesperadin (10× IC_50_)^17^. Parasite cell cycle progression under Hesperadin pressure was monitored firstly with flow cytometry (as described in^17^) (Supplementary Fig. 1) using SYBR Green I nuclear staining. Fluorescence was collected in the FL-1 channel (FITC signal) on a Becton Dickinson AccuriTM C6 Plus cytometer. A minimum of 100,000 events were captured in each instance and analysed using FlowJo version 10.1 software. Parasite viability, nuclear content and morphology were evaluated by fluorescence microscopy with a 1:1000 dilution of either propidium iodide viability staining (emission 617 nm, excitation 536 nm), Hoechst 33258 or SYBR Green I nuclear staining (emission 478 and 530 nm, excitation 345 and 497 nm, respectively) in the dark for 15 min. Direct immunofluorescence imaging was performed on live and fixed parasites (4% paraformaldehyde, 0.025% glutaraldehyde, 30 min) on poly-Lys-coated coverslips, permeabilized with 0.1% Triton X-100, followed by exposure to 1 : 500 dilution AlexaFluor 647-conjugated rabbit anti-α-tubulin for 45 min in the dark. Imaging was performed on a Zeiss LSM 880 Inverted Confocal Laser Scanning Microscope (LSM) - Airyscan detector (Zeiss, Germany) for super-resolution imaging in the appropriate channels with a ×100 oil-immersion objective and 1.4 numerical aperture. Photomultiplier gain was set to 600 V and laser scanning times were kept at 45 s. Zeiss ZEN lite blue edition software (Zeiss, Germany) was used for initial digital image processing and ImageJ version 1.52d open source software (NIH, USA) was used for fluorescence intensity analysis as grey values in pixels.

### Resistant mutant selection

Two *P. falciparum* strains with different spontaneous mutations rates were chosen, the drug-sensitive reference strain 3D7 and the multi-drug-resistant Dd2. A pure clonal line was derived from each strain by limiting dilution to reduce genetic variation before resistance selection. One clone was chosen and 1 ml aliquots frozen away. An aliquot was expanded to generate the ~8 × 10^8^ parasites used for resistance selection, which were equally divided among four T25 flasks in 10 ml of complete RPMI-1640 medium, containing a concentration of Hesperadin equal to tenfold its IC_50_ for the strains used. Flasks were gassed with a 1% oxygen, 5% carbon dioxide, 94% nitrogen mixture, sealed and incubated at 37 °C. The growth medium was changed every 2 days maintaining the selection pressure and half the RBCs were replaced with fresh cells every week. Parasite growth was monitored microscopically weekly for the first month and every 2 days thereafter. This regime was continued until visible parasite growth appeared in the flasks. There was an end-of-experiment control flask incubated under the same conditions with 10× the IC_50_ of pyrimethamine. This antimalarial has a known low frequency of target-based, spontaneous resistance mutations^43^. No parasites were observed in this control culture before ring stages were detected in the flask containing Hesperadin.

When Hesperadin-resistant cell growth was observed, samples were subcultured in drug-free medium for a week (approximately three cell cycles) to enrich for stable resistant cells and the IC_50_ for Hesperadin measured in the bulk culture. Single clones were then isolated by limiting dilution in the presence of the drug. Two clones were retained from each flask to be used as duplicates and their individual IC_50_ for Hesperadin was determined, but only isolates from different flasks were considered independent mutants. A flow diagram depicting the above steps is shown in Supplementary Fig. 2.

### Whole-genome sequencing and analysis

Sequencing libraries were prepared from DNA extracted from the mutant and parental cell lines with the Nextera XT kit (Illumina), using the standard dual index protocol. They were sequenced on the Illumina HiSeq 2500 in RapidRun mode, with an average read length of 100 base pairs. Reads were aligned to the *P. falciparum* 3D7 reference genome (PlasmoDB v. 13.0), as previously described^44^, with single nucleotide variants (SNVs) and insertion/deletions (INDELs) called with the Genome Analysis Toolkit’s (GATK) HaplotypeCaller^45–47^. SNVs were removed if they met the following criteria: ReadPosRankSum > 8.0 or < −8.0, QUAL < 500, Quality by Depth (QD) < 2, Mapping Quality Rank Sum < −12.5, and filtered depth (DP) < 7. Indels were removed if they met the following criteria: ReadPosRankSum < −20, QUAL < 500, QD < 2, and DP < 7. Furthermore, we removed mutations where read coverage was <5 and/or where mixed read ratios were >0.2 (reference/total reads) across all samples. Variants were annotated using SnpEff^48^. Identified variants were then compared between the resistant clones and the parent clone. Since all parasite lines were cloned before sequencing, only homozygous variant calls were retained.

As the *P. falciparum* genome is 90–95% AT-rich in intergenic regions, only coding regions were analyzed. For CNV analysis, average coverage of each gene was calculated with GATK’s DiagnoseTargets tool, supplying gene lengths and locations as the analysed intervals. The hypervariable var, rifin and stevor gene families were removed. The data were then normalized for read coverage relative to the parent clone. The data were visually inspected to identify copy number variations (CNVs). Mutations described in this work were confirmed by targeted sequencing.

### Molecular cloning, protein expression and kinase assays of PfNek1 variants

Site-directed mutagenesis was performed on a previously constructed GST-tagged Pfnek1 plasmid^25^, carrying the catalytic domain of the PfNek1 ORF (residues 1–432) in the pGEX-3X vector. The following mutants of PfNek1 were constructed and checked by DNA sequencing: (a) methionine to isoleucine change at residue 66 (M66I); (b) glycine to aspartic acid change at residue 143 (G143D); and (c) alanine to glycine change at residue 246 (A246G). The same plasmid carrying a catalytically inactive PfNek1 with a serine to alanine change at residue 200 (S200A) had been previously constructed and characterized^25^. The PfNek1 truncation plasmid (pCAM-PfNek1) was generated by inserting a PCR product corresponding to 1027 bp of Pf*nek1* sequence immediately 5′ to the S854STOP mutation into the pCAM-BSD-HA vector^26,27^, which contains a cassette conferring resistance to blasticidin. The PCR product was obtained using 3D7 genomic DNA as template and the following oligonucleotides: forward, 97J 5′-GGGCTGCAGagaatggatagacttgaaaga-3′; reverse, 98J 5′-GGGGGATCCtttccttctgctcacattgtt-3′, which contain PstI and BamHI sites (underlined), respectively. Transfectants were analysed by PCR using appropriate primer pairs. Primers 490 (5′-GATGAAGAGGGAAATATACG-3′) and 41J (5′-GGGGGATCCtctacaggtatataaacccct-3′) produced a 2.1 kb fragment corresponding to the undisrupted Pf*nek1* locus. The primer pair 167 (5′-TATTCCTAATCATGTAAATCTTAAA-3′) and 275 (5′-CGAACATTAAGCTGCCATATCC-3′), specific for the pCAM-BSD-HA vector, produced a 1.0 kb fragment corresponding to the plasmid. Primer pairs 490/275 and 167/41J amplified across the 5′- and 3′-ends of the integration site, giving rise to 1.3 and 1.7 kb products, respectively.

Expression and purification of wild-type and mutant GST-PfNek1 fusion proteins were performed as described^25^. Kinase assays were carried in a final reaction volume of 30 μl, containing 25 mM Tris-HCl pH 7.5, 20 mM MgCl_2_, 2 mM MnCl_2_, 10 µM ATP, 2.5 μCi [γ-^32^P]ATP and 5 μg of myelin basic protein (MBP) (Sigma). Reactions were initiated by the addition of 1 μg of the purified recombinant wild-type or mutated GST-PfNek1. Increasing concentrations of Hesperadin (up to 1 µM) and Purvalanol B (up to 10 µM) in DMSO were added to determine their effect on kinase activity. The reaction was left to proceed for 30 min at 30 °C and was stopped by the addition of Laemmli sample buffer and boiling for 3 min. Reaction products were analysed on 4–12% gradient SDS-polyacrylamide gels. Following Coomassie blue staining, the gels were dried and ^32^P-labelled products were detected using a BAS Storage Phosphor Screen (GE Healthcare) for visualization on a Typhoon Trio Imager (GE Healthcare).

### Parasite transfection and Southern blot

Cultures synchronized to 5–8% ring-stage parasitaemia after sorbitol treatment were used for DNA transfections. Plasmids were transfected into parasites suspended in Cytomix solution (120 mM KCl, 150 µM CaCl_2_, 10 mM K_2_HPO_4_/KH_2_PO_4_, 25 mM HEPES, 2 mM EGTA, 5 mM MgCl_2_ pH 7.6) (Sigma-Aldrich) by electroporation in a Gene Pulser Xcell (0.31 kV, 950 µF) (Bio-Rad). After electroporation, parasites were resuspended in 2 ml of complete RPMI and transferred to a T25 culture flask containing 8 ml of complete RPMI-1640, adjusted to 4% haematocrit, gassed and incubated at 37 °C. Approximately 2 h post transfection, media was replaced with 9.5 ml of fresh complete RPMI and drug selection was applied for the resistance marker in the transfected plasmid the following day.

For the Southern blot in Fig. 6c, 2.5 μg of parasite genomic DNA and 1 ng of pCAM-Pfnek1 plasmid DNA were digested with EcoRV, separated on a 1% agarose gel and transferred to a nylon membrane. The blot was probed with the digoxigenin (DIG)-labelled Pfnek1 sequence amplified from 3D7 genomic DNA with the primer pair 97J/98J (used to produce the pCAM-Pfnek1 truncation construct) and incubated with an anti-DIG antibody conjugated to alkaline phosphatase (Roche). Chemoluminescence detection was performed using CDP-star Chemiluminescence substrate (Roche) and imaged using a Bio-Rad ChemiDoc XRS + imaging system.

### Statistics and reproducibility

Numerical IC_50_ values shown on tables are averages of three independent biological experiments (i.e., three different parasite cultures), each performed in triplicate (i.e., three multiwell plates per culture). Each of the three averaged values was obtained by least squares fitting of a three-parameter curve in GraphPad Prism 6.0.1. Flow cytometry histograms were obtained from the analysis of 100,000 events, applying the gating conditions shown in Supplementary Fig. 1 and using the FlowJo 10.1 software.

### Reporting summary

Further information on research design is available in the Nature Research Reporting Summary linked to this article.

## Supplementary information

Supplementary Information

Description of Additional Supplementary Files

Supplementary Data 1

Reporting Summary

## Data Availability

Sequencing files were deposited in the National Center for Biotechnology Information (NCBI) Sequence Read Archive (SRA) database with accession code PRJNA598363.
