# Supplementary material for: Human Aurora kinase inhibitor Hesperadin reveals epistatic interaction between Plasmodium falciparum PfArk1 and PfNek1 kinases
Source: Commun Biol. 2020 Nov 20;3:701. doi: 10.1038/s42003-020-01424-z (PMC7679417; doi:10.1038/s42003-020-01424-z)
Supplement: Supplementary file 1 — Supplementary Information [file 42003_2020_1424_MOESM1_ESM.pdf]

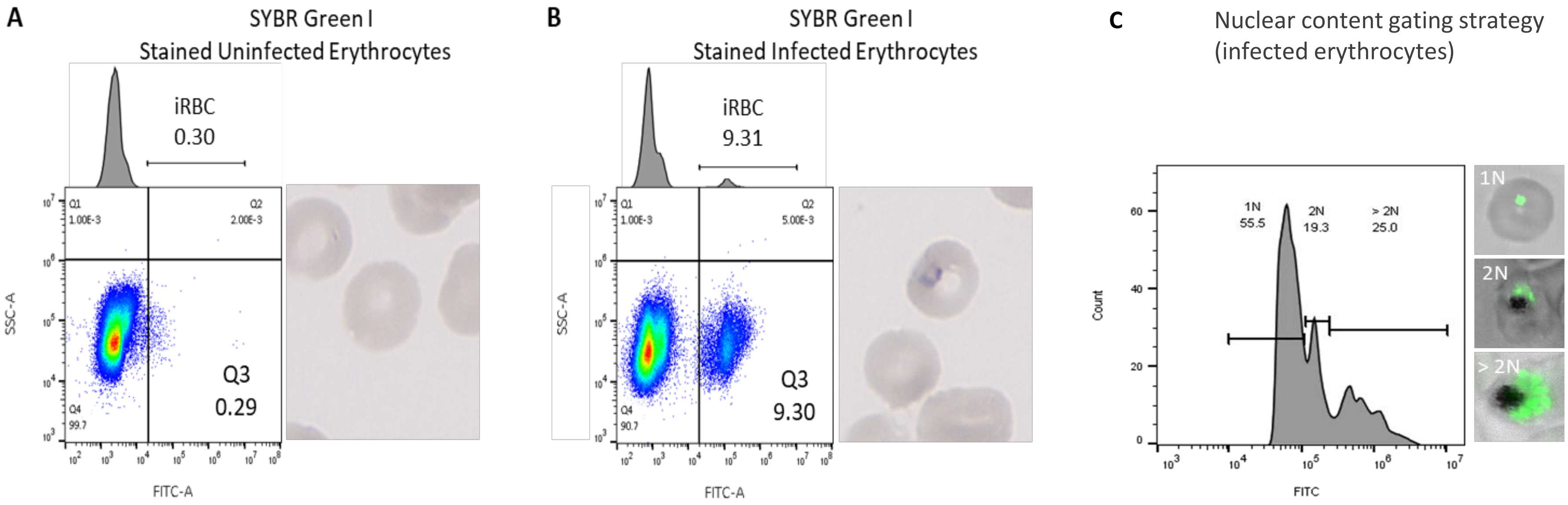

Supplementary Figure 1: Flow cytometry gating strategy used for intraerythrocytic *P. falciparum* parasite life cycle progression.
(A) Density plot represents the side scatter (SSC) of the SYBR Green I stained uninfected erythrocytes. The bar indicated on the corresponding histograms (iRBC) corresponds to the gate set to differentiate between SYBR Green I stained uninfected erythrocytes and infected erythrocytes fluorescence. **(B)** Gating conditions were applied to SYBR Green I stained intraerythrocytic *P. falciparum* 3D7 parasites. Density plots and histograms were generated using FlowJo version 10.1 software. Parasitaemia was confirmed through Giemsa stained light microscopy at 1000x magnification. (C) Histogram represents parasite DNA copy number (N) with corresponding parasite morphology; ring-stage parasites (1N), trophozoite-stage parasites (2N), schizont-stage parasites (> 2N). In all cases, a total of 100 000 events were captured. Histograms were generated using FlowJo version 10.1 software. Morphology was confirmed through SYBR Green I confocal fluorescence microscopy using a Zeiss LSM 880 Confocal Laser Scanning Microscope (LSM).

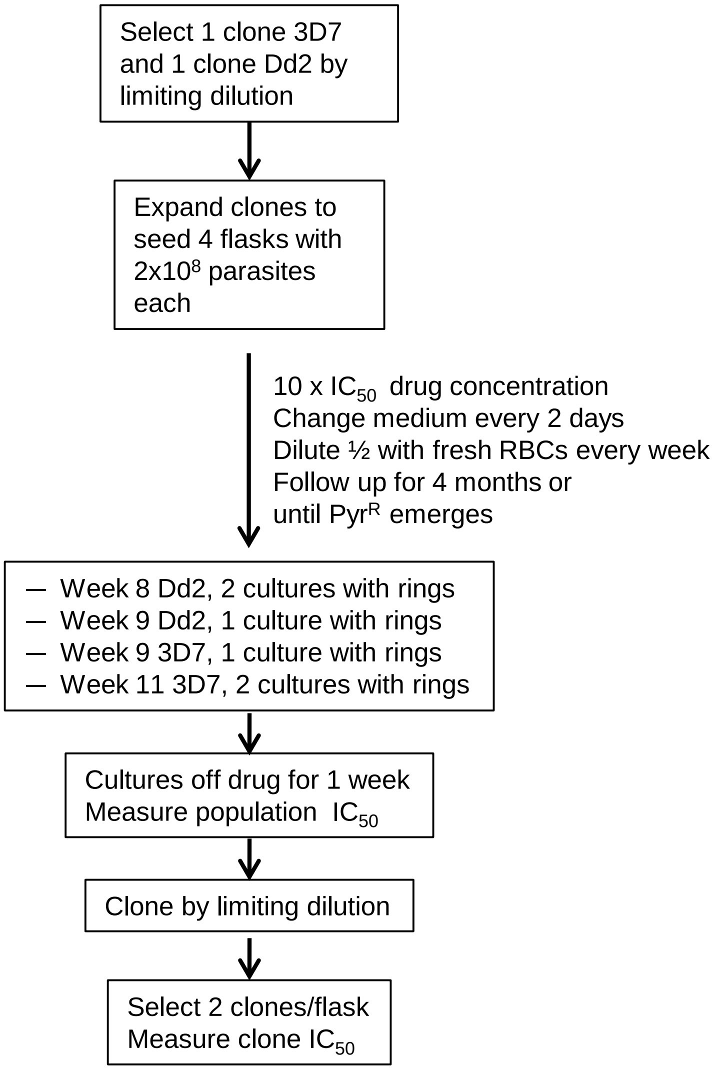

Supplementary Fig. 2: Selection scheme for Hesperadin-resistant mutants of *Plasmodium falciparum* strains 3D7 and Dd2

Supplementary Fig 3: Full-size gel and autoradiogram for panels **c** and **d** in Fig. 5

Panel 5c

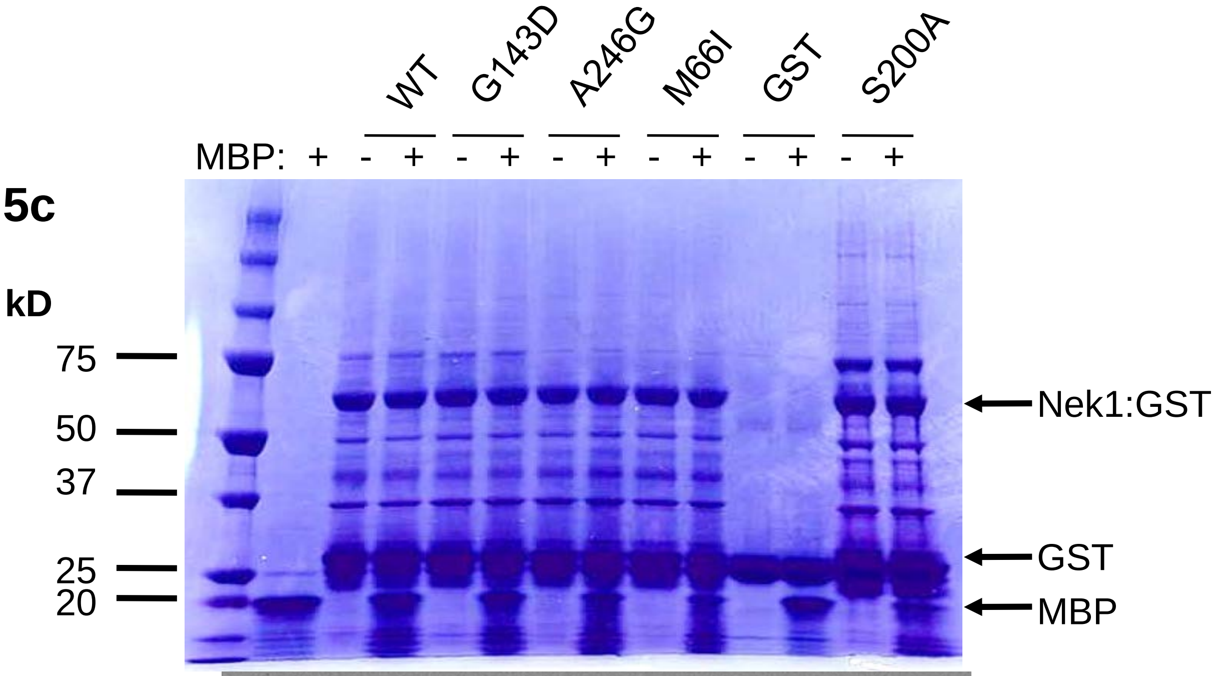

Panel 5d

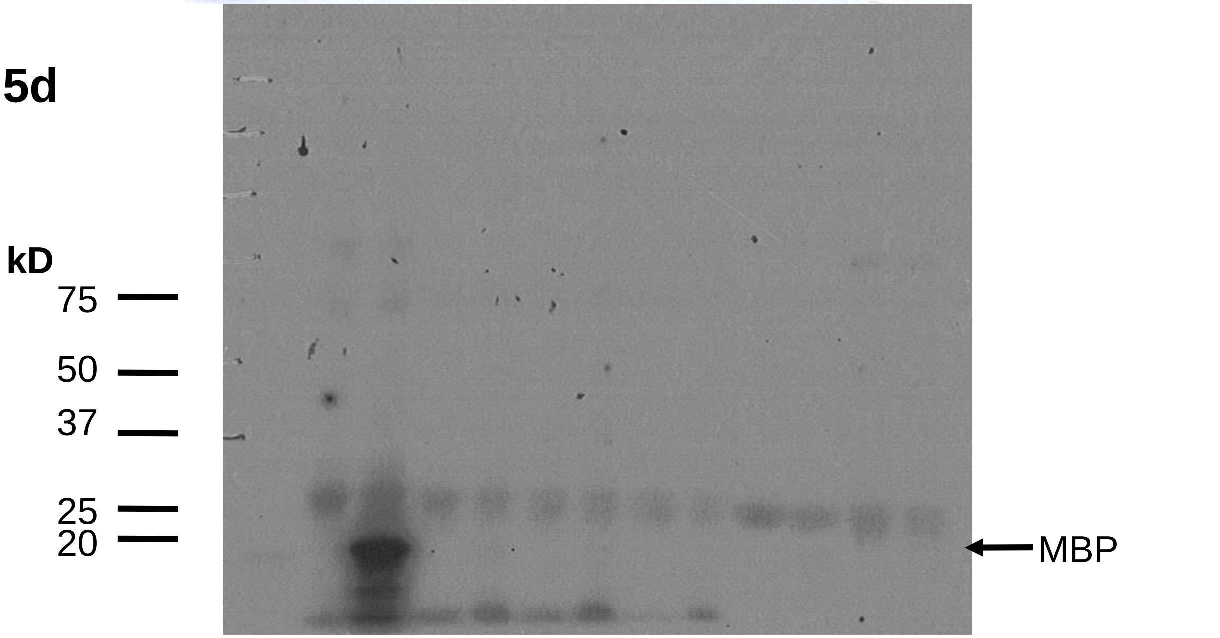

Supplementary Table 1. Activity of different Aurora kinase inhibitors against *Plasmodium falciparum*

Compound	Main human target	Activity against human AurK (IC_{50} nM)	Reference	Activity against *P. falciparum* (IC_{50} nM)	
				3D7	Dd2
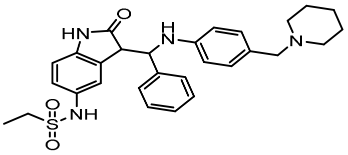 TCMDC-135395 Hesperadin	AurB	250	1	40	40
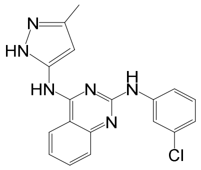 TCMDC-134695	AurA+AurB	NA	NA	244	1454
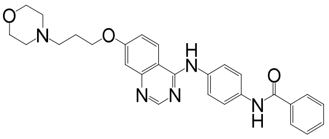 TCMDC-125873 ZM-447439	AurA+AurB	110+130	2	161	342
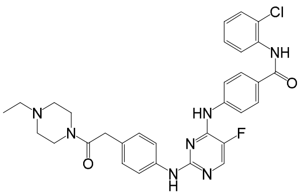 S1451	AurA	3	3	430	448
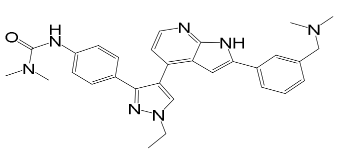 GSK1070916	AurB+ AurC	4+7	4	750	174
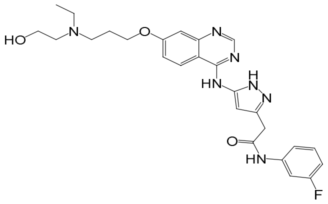 Barasertib	AurB	1	5	365	340
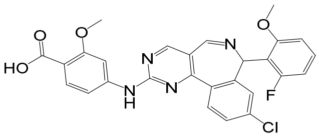 Alisertib	AurA	1	6	10260	3245

NA: not available

References

1. Hauf, S. et al. The small molecule Hesperadin reveals a role for Aurora B in correcting kinetochore-microtubule attachment and in maintaining the spindle assembly checkpoint. *J. Cell Biol.* **161**, 281–294 (2003).
2. Ditchfield, C. et al. Aurora B couples chromosome alignment with anaphase by targeting BubR1, Mad2, and Cenp-E to kinetochores. *J. Cell Biol.***161**, 267–280 (2003).
3. Aliagas-Martin, I. et al. A class of 2,4-bisanilinopyrimidine Aurora A inhibitors with unusually high selectivity against Aurora B. *J. Med. Chem.* **52**, 3300–3307 (2009).
4. Anderson, K. et al. Biochemical characterization of GSK1070916, a potent and selective inhibitor of Aurora B and Aurora C kinases with an extremely long residence time. *Biochem. J.* **420**, 259–265 (2009).
5. Yang, J. et al. AZD1152, a novel and selective aurora B kinase inhibitor, induces growth arrest, apoptosis, and sensitization for tubulin depolymerizing agent or topoisomerase II inhibitor in human acute leukemia cells in vitro and in vivo. *Blood* **110**, 2034–2040 (2007).
6. Manfredi, M. G. et al. Characterization of Alisertib (MLN8237), an investigational small-molecule inhibitor of aurora A kinase using novel in vivo pharmacodynamic assays. *Clin. Cancer Res.* **17**, 7614–7624 (2011).

Supplementary Table 2. Results from Illumina whole genome sequencing.

Genetic background	Clone	Total nr. of reads	*P. falciparum* aligned reads (%)	Genome fraction covered by 5 or more reads (%)	Mean coverage (%)
3D7	Parent	23133022	23074916 (99.7)	99.5	86.7
	6G7	23454952	23417709 (99.8)	99.6	88.8
Dd2	Parent	24328716	23809521 (97.8)	97	82.4
	8E6	18016686	18049079 (98.5)	96.8	68.2
	1F9	27175908	26659935 (96.7)	96.7	92.34
	5G8	24516332	24197401 (98.7)	97.1	83.8
