# Supplementary material for: Human Aurora kinase inhibitor Hesperadin reveals epistatic interaction between Plasmodium falciparum PfArk1 and PfNek1 kinases
Source: Commun Biol. 2020 Nov 20;3:701. doi: 10.1038/s42003-020-01424-z (PMC7679417; doi:10.1038/s42003-020-01424-z)
Supplement: Supplementary file 2 — Description of Additional Supplementary Files [file 42003_2020_1424_MOESM2_ESM.pdf]

Description of Additional Supplementary Files

File Name: Supplementary Data 1
Description: Numerical data used to build the plots in figures 1a, 2b, 3 and 7.
