# Supplementary material for: Human Aurora kinase inhibitor Hesperadin reveals epistatic interaction between Plasmodium falciparum PfArk1 and PfNek1 kinases
Source: Commun Biol. 2020 Nov 20;3:701. doi: 10.1038/s42003-020-01424-z (PMC7679417; doi:10.1038/s42003-020-01424-z)
Supplement: Supplementary file 4 — Reporting Summary [file 42003_2020_1424_MOESM4_ESM.pdf]

nature research

Corresponding author(s): Jose F. Garcia-Bustos

Last updated by author(s): Sep 21, 2020

Reporting Summary

Nature Research wishes to improve the reproducibility of the work that we publish. This form provides structure for consistency and transparency in reporting. For further information on Nature Research policies, see Authors & Referees and the Editorial Policy Checklist.

Statistics

For all statistical analyses, confirm that the following items are present in the figure legend, table legend, main text, or Methods section.

n/a	Confirmed	
☐	☒	The exact sample size (*n*) for each experimental group/condition, given as a discrete number and unit of measurement
☐	☒	A statement on whether measurements were taken from distinct samples or whether the same sample was measured repeatedly
☐	☒	The statistical test(s) used AND whether they are one- or two-sided<br>*Only common tests should be described solely by name; describe more complex techniques in the Methods section.*
☒	☐	A description of all covariates tested
☒	☐	A description of any assumptions or corrections, such as tests of normality and adjustment for multiple comparisons
☐	☒	A full description of the statistical parameters including central tendency (e.g. means) or other basic estimates (e.g. regression coefficient) AND variation (e.g. standard deviation) or associated estimates of uncertainty (e.g. confidence intervals)
☒	☐	For null hypothesis testing, the test statistic (e.g. *F*, *t*, *r*) with confidence intervals, effect sizes, degrees of freedom and *P* value noted<br>*Give P values as exact values whenever suitable.*
☒	☐	For Bayesian analysis, information on the choice of priors and Markov chain Monte Carlo settings
☒	☐	For hierarchical and complex designs, identification of the appropriate level for tests and full reporting of outcomes
☒	☐	Estimates of effect sizes (e.g. Cohen's *d*, Pearson's *r*), indicating how they were calculated

Our web collection on statistics for biologists contains articles on many of the points above.

Software and code

Policy information about availability of computer code

Data collection	*Provide a description of all commercial, open source and custom code used to collect the data in this study, specifying the version used OR state that no software was used.*
Data analysis	FlowJo software v.10.1; Genome Analysis Toolkit's (GATK) HaplotypeCaller v. 3.4; GraphPad Prism v.6.01

For manuscripts utilizing custom algorithms or software that are central to the research but not yet described in published literature, software must be made available to editors/reviewers. We strongly encourage code deposition in a community repository (e.g. GitHub). See the Nature Research guidelines for submitting code & software for further information.

Data

Policy information about availability of data

All manuscripts must include a data availability statement. This statement should provide the following information, where applicable:

- Accession codes, unique identifiers, or web links for publicly available datasets
- A list of figures that have associated raw data
- A description of any restrictions on data availability

NCBI Sequence Read Archive (SRA) database accession code PRJNA598363

Field-specific reporting

Please select the one below that is the best fit for your research. If you are not sure, read the appropriate sections before making your selection.

☒ Life sciences ☐ Behavioural & social sciences ☐ Ecological, evolutionary & environmental sciences

Life sciences study design

All studies must disclose on these points even when the disclosure is negative.

Sample size	N/A
Data exclusions	P. falciparum hypervariable var, rifin and stevor gene families were removed from the analysis
Replication	Biochemical and cellular measurements were made on three biologically independent experiments, each with three technical replicates.
Randomization	N/A
Blinding	N/A

Reporting for specific materials, systems and methods

We require information from authors about some types of materials, experimental systems and methods used in many studies. Here, indicate whether each material, system or method listed is relevant to your study. If you are not sure if a list item applies to your research, read the appropriate section before selecting a response.

Materials & experimental systems

n/a	Involved in the study	
☐	☒	Antibodies
☒	☐	Eukaryotic cell lines
☒	☐	Palaeontology
☒	☐	Animals and other organisms
☒	☐	Human research participants
☒	☐	Clinical data

Methods

n/a	Involved in the study	
☒	☐	ChIP-seq
☐	☒	Flow cytometry
☒	☐	MRI-based neuroimaging

Antibodies

Antibodies used	AlexaFluor 594 conjugated anti-α-tubulin (BioLegend)
Validation	Mouse monoclonal against full length recombinant human alpha tubulin. The 10D8 antibody recognizes α-tubulin in all species and is useful for Western blotting, and immunofluorescence staining.

Flow Cytometry

Plots

Confirm that:

☒ The axis labels state the marker and fluorochrome used (e.g. CD4-FITC).

☒ The axis scales are clearly visible. Include numbers along axes only for bottom left plot of group (a 'group' is an analysis of identical markers).

☒ All plots are contour plots with outliers or pseudocolor plots.

☒ A numerical value for number of cells or percentage (with statistics) is provided.

Methodology

Sample preparation	P. falciparum 3D7 or NF54 in vitro cultures
Instrument	Becton Dickinson AccuriTM C6 Plus cytometer
Software	FlowJo software v.10.1
Cell population abundance	RBCs with 1% P. falciparum parasitemia by microscopic manual counting. Parasites are the only nucleated cells in the sample.
Gating strategy	Dataset cleansing was done by removing instrument induced fluorescence anomalies, doublets and/or cell clumps, cell debris and any unwanted events. Primary gating was performed manually based on control samples; an unstained uninfected erythrocyte sample and a stained uninfected erythrocyte sample. Background DNA-free erythrocyte fluorescence signal was deducted using the latter control sample. Primary gating was performed to separate parasite infected erythrocytes (populations

with nucleus) from the uninfected erythrocytes, as a measure of parasitaemia. The parasite infected erythrocyte population was used to perform secondary gating; segregate parasite infected erythrocytes according to their DNA copy number. Ring and early trophozoite parasites with a single nucleus corresponded to 1N DNA copy number, while mature trophozoite and schizont parasites contained multiple nuclei, 2N and >2N, respectively.

☒ Tick this box to confirm that a figure exemplifying the gating strategy is provided in the Supplementary Information.
